# Substantial bulk photovoltaic effect enhancement via nanolayering

**DOI:** 10.1038/ncomms10419

**Published:** 2016-01-21

**Authors:** Fenggong Wang, Steve M. Young, Fan Zheng, Ilya Grinberg, Andrew M. Rappe

**Affiliations:** 1 The Makineni Theoretical Laboratories, Department of Chemistry, University of Pennsylvania, Philadelphia, Pennsylvania 19104-6323, USA; 2 Center for Computational Materials Science, United States Naval Research Laboratory, Washington, DC 20375, USA

## Abstract

Spontaneous polarization and inversion symmetry breaking in ferroelectric materials lead to their use as photovoltaic devices. However, further advancement of their applications are hindered by the paucity of ways of reducing bandgaps and enhancing photocurrent. By unravelling the correlation between ferroelectric materials' responses to solar irradiation and their local structure and electric polarization landscapes, here we show from first principles that substantial bulk photovoltaic effect enhancement can be achieved by nanolayering PbTiO_3_ with nickel ions and oxygen vacancies ((PbNiO_2_)_*x*_(PbTiO_3_)_1−*x*_). The enhancement of the total photocurrent for different spacings between the Ni-containing layers can be as high as 43 times due to a smaller bandgap and photocurrent direction alignment for all absorption energies. This is due to the electrostatic effect that arises from nanolayering. This opens up the possibility for control of the bulk photovoltaic effect in ferroelectric materials by nanoscale engineering of their structure and composition.

## Abstract (web summary)

Spontaneous polarization in ferroelectric materials leads to their use as photovoltaic devices. Here, the authors show by first-principles calculations how nanolayering of PbTiO_3_ with nickel ions and oxygen vacancies can result in enhanced photocurrents due to smaller bandgaps and photocurrent alignment.

## Graphical abstract

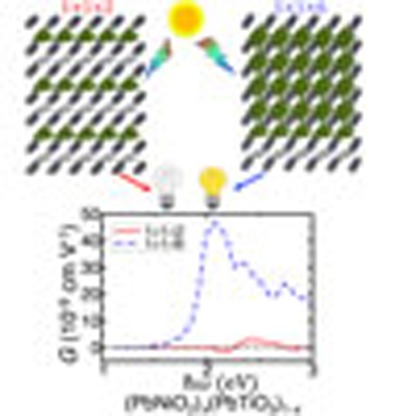

Studies of photo-induced effects in ferroelectrics have experienced a revival due to the demonstration of a variety of fascinating physical effects, and in particular an increase in the power conversion efficiency of ferroelectric-based solar cells^1^,^2^,^3^,^4^,^5^. In particular, recent experiments on BiFeO_3_ thin films with absorption at the visible-light edge have revealed phenomena that were not observed in classic studies of bulk ferroelectric photoresponses^6^,^7^,^8^,^9^. Advances in the power conversion efficiency of ferroelectric-based solar cells and the discovery of visible-light-absorbing ferroelectric materials have provided further encouragement for this field^3^,^4^,^10^,^11^.

The recent experimental discoveries and progress in thin film fabrication have been accompanied by significant advances in the understanding of the unique light–matter interactions enabled by the presence of a spontaneous, switchable polarization in ferroelectric materials. In particular, it has been demonstrated that the very large open-circuit photovoltage observed in BiFeO_3_ films is due to the bulk photovoltaic effect (BPVE)^12^,^13^,^14^,^15^,^7^; in turn, BPVE has been shown to arise from the shift current mechanism, in which electrons are continuously excited under sustained illumination to quasiparticle coherent states that have intrinsic momenta, generating a spontaneous direct short-circuit photocurrent^16^,^17^. While the fundamental mechanism underlying BPVE has been determined, it is not yet completely understood how the BPVE depends on the structure of ferroelectric materials. In particular, a thorough understanding of how the BPVE can be controlled through adjustment of the atomic and electronic compositions and change of the chemical bonding properties is highly desired from the fundamental standpoint as well as for future development of BPVE-based devices.

Nano heterostructured materials have been shown to exhibit intriguing physics that are inaccessible in their bulk material counterparts due to the quantum confinement effect, the sensitivity of functional properties to structure, as well as the unique properties at interfaces, such as in the case of the two-dimensional electron gas at the LaAlO_3_/SrTiO_3_ interface^18^. Furthermore, it has been established that there is a connection between materials' local chemistry and structural properties, their fundamental and functional properties, and their responses to external stimuli^19^,^20^. Therefore, superior properties, specifically an enhancement of the photovoltaic performance, may be obtained by tuning the local structure and the landscape of local electric polarization in oxides with an engineered bandgap^21^. Motivated by the strong effect of electrostatics on materials' bandgaps and electronic transitions^22^,^23^, here we explore the possibility of nano-ordering photoresponsive ferroelectric materials to control and enhance the BPVE, highlighting the importance of length scale (layer thickness), electronics and electrostatics for photovoltaic performance.

We use first-principles calculations to investigate the relationship between local displacements, electronic structure and the BPVE response in the nanolayered Ni+vacancy-substituted PbTiO_3_ material Pb(Ni_*x*_Ti_1−*x*_)O_3−*x*_ (called (PbNiO_2_)_*x*_(PbTiO_3_)_1−*x*_ or PNT for brevity)^24^,^25^. We choose Ni because it is a late-transition metal, and so it is relatively electronegative, with filled and empty *d* orbitals near in energy; accordingly, Ni substitution induces a low-lying conduction band (CB). The combination of Ni and vacancy substitution guarantees that Ni ions are found in the +2 charge states. The Ni^2+^ state is also preferred to the Ni^3+^ state, because Ni^3+^ ion has a small ionic radius (0.60 Å) and is only stable in perovskites with small La^3+^ ions on the *A* site, whereas the larger Ni^2+^ ion is known to be stable in ferroelectric compounds^26^. Because it is more computationally convenient, we choose PNT to serve as a prototype for the recently developed KBNNO material that for the first time showed excellent light absorption in the visible range by an oxide ferroelectric, breaking the decades-long restriction of ferroelectric oxides to only near-ultraviolet and ultraviolet absorption^10^. PNT is also closely related to the Ni-doped Pb(Zr_1−*x*_Ti_*x*_)O_3−*x*_ (PZT) material (PNZT) synthesized in thin film form and shown to exhibit improved light-absorption properties relative to the parent PZT material^27^. We show that the BPVE response over a wide range of frequencies is sensitive to the changes in the electronic structure that are driven by changes in the local cation displacements. The PNT nanostructures exhibit several unexpected and counterintuitive effects that enable the tuning of the absorption properties and a marked enhancement (by a factor of 43) of the BPVE response by nanoscale (1–2 nm) layering. Our results suggest that nanoscale control of composition and structure is an attractive route for manipulating the BPVE in ferroelectric oxides.

## Results

### Oxygen vacancy and cation arrangement

We start from exploring the relative stabilities of different O vacancy sites by choosing a 1 × 1 × 2 supercell with one Ti atom substituted by Ni (Ni_Ti_) and one O atom removed (*V*_O_), corresponding to the (PbNiO_2_)(PbTiO_3_) composition (*x*=1/2). Total energy analysis indicates that O vacancies prefer to form adjacent to Ni (Supplementary Fig. 1), because this provides more effective charge compensation. In addition, under normal conditions the equatorial O vacancies are more stable than the apical O vacancies. However, the apical O vacancies become more stable by 29 meV per atom with 1% in-plane compressive strains. Furthermore, the apical O vacancies are preferred in pure PbTiO_3_ (ref. 28), and it is likely that a large proportion of the apical O vacancies are preserved after Ni incorporation.

The location of O vacancy can significantly affect the polarity of the structure and the composition of the contributing electronic states, and in turn may also affect the photocurrent. The shift current response of the 1 × 1 × 2 supercell with equatorial O vacancies is almost negligible. This is because when equatorial O vacancies are introduced, the relaxed structure with NiO_4_ complexes in the vertical *yz* (or *xz*) plane is nearly centrosymmetric, whereas the presence of apical O vacancies enlarges the lattice asymmetry. While emphasizing that a larger lattice asymmetry does not always induce a larger shift current, the obtained structural change is nevertheless consistent with the shift current results.

The *x*=1/2 composition may also take the rock salt *B*-cation arrangement (Supplementary Fig. 1), in which the O vacancy has no preferred site. Even though the rock salt cation arrangement is less energetically favourable than its layered counterpart, it exhibits a more substantial photocurrent response (Table 1). Comparison of their total and projected densities of states shows that the Pb 6*p* orbitals contribute the most to the bottom of the CB in the rock salt structure, but in the layered structure the Ni 3*d* orbitals contribute the most (Supplementary Fig. 1). This occurs because in the rock salt structure the Ni–*V*_O_–Ni network is interrupted by the Ti and Pb atoms, making the Ni 3*d* orbitals mainly localized at an energy position of 1 eV higher than the bottom of the CB. Electronic transitions involving the more delocalized Pb 6*p* oribitals facilitate the motion of the photocurrent carriers and lead to stronger photocurrent response.

### Photocurrent cancellation in PNT

**Figure 1:**
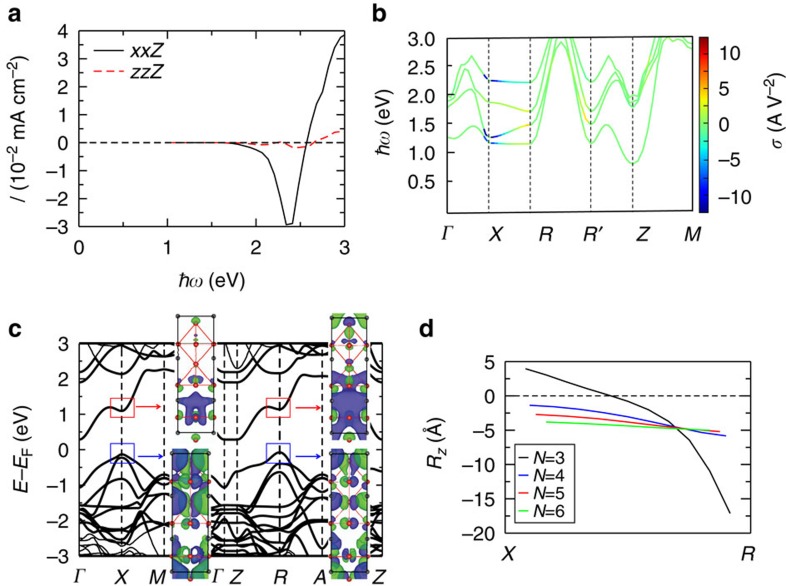
The photocurrent cancellation in layered PNT and its electronic and wavefunction origins. The (**a**) photocurrent (assuming 0.1 W cm^−2^ light absorption), (**b**) *k*-resolved photocurrent and (**c**) band structure and real-space wavefunction isosurfaces corresponding to the states indicated by the rectangular regions, of the 1 × 1 × 3-layered PNT. In **b**, the colour gives the value of the photocurrent response *σ* (A V^−2^). The near-gap response is dominated by the region around the line from *X* to *R*, and changes direction along this path. 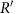 indicates *R* plus a reciprocal lattice vector, so that 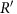 traverses the Brillouin zone. (**d**) The shift vector for the *k* points along the *X*–*R* line in the Brillouin zone of the 1 × 1 × *N*-layered PNT.

Inspection of the shift current results (Fig. 1a; we focus only on the most substantial, *xxZ* component) for the PbTiO_3_ with 33% Ni+vac composition (1 × 1 × 3 superlattice) shows that the overall current produced by illumination in the visible range is small, due to a strong cancellation of the contributions for light absorption of photons in the 2–2.5 eV range and in the 2.5–3.0 eV range. Such a cancellation was also found by our theoretical calculations for the KBNNO material^29^. This is unlike the shift currents calculated for PbTiO_3_ and BiFeO_3_ that exhibit a uniform sign of the photoresponse throughout the photon spectrum. It is likely that the shift current cancellation observed in KBNNO and PNT is related to the greater diversity of electronic orbital character caused by the presence of dissimilar Ni and Ti/Nb local environments. Examination of the *k*-resolved shift current (Fig. 1b) shows that the electronic transitions occurring near the *X*(0, 0.5, 0) and *R*(0, 0.5, 0.5) *k* points and along the *X*–*R* line induce the most substantial photocurrent responses and are thus the most important for the band-edge electronic transitions. However, due to the changes in the direction of the shift vector (Fig. 1d), the contributions from the transitions at *X* and at *R* largely counteract each other. This suggests that the BPVE response can be significantly increased by aligning the sign of the photocurrent throughout the Brillouin zone.

To elucidate the origin of the photocurrent cancellation, we consider the details of the electronic states at the *X* and *R* points. The calculated PNT band structure (Fig. 1c) shows that the bandgap is reduced due to the introduction by nickel of a low-lying CB into the bulk PbTiO_3_ gap; as a result, the band-edge electronic transitions, which will be responsible for the shift current response generated by the visible light, occur only between the valence bands and this particular CB. Real-space wavefunction distribution analysis for the highest valence band shows that it is essentially delocalized at both *X* and *R*, with the wavefunction extending to the whole supercell because of the overlap among the Pb 6*s*, Ti 3*d*, Ni 3*d* and O 2*p* orbitals. However, the wavefunction distribution for the lowest CB shows substantial differences between *X* and *R*. At *X*, it arises mainly from the Ni 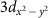 and the nearby Pb 6*p* orbitals, and is more localized, whereas at *R* it becomes much more delocalized and has larger orbital contributions from the Pb 6*p*, Ti 3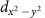 and Ti 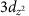 orbitals. The greater delocalization at the *R* point is directly related to the wavefunction character; compared with the Ni 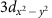 orbital, the Pb 6*p* and Ti 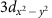 orbitals are more delocalized, while the Ti 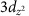 orbitals extend the wavefunction along the photocurrent direction. The increasing delocalization of the CB minimum (CBM) wavefunction from *X* to *R* is accompanied by the change in the sign of the shift current and leads to a bigger shift vector magnitude (shift vector can be viewed as an indicator of the nonthermal carrier mobility) (Fig. 1d).

### Photocurrent enhancement by artificial manipulation

**Figure 2:**
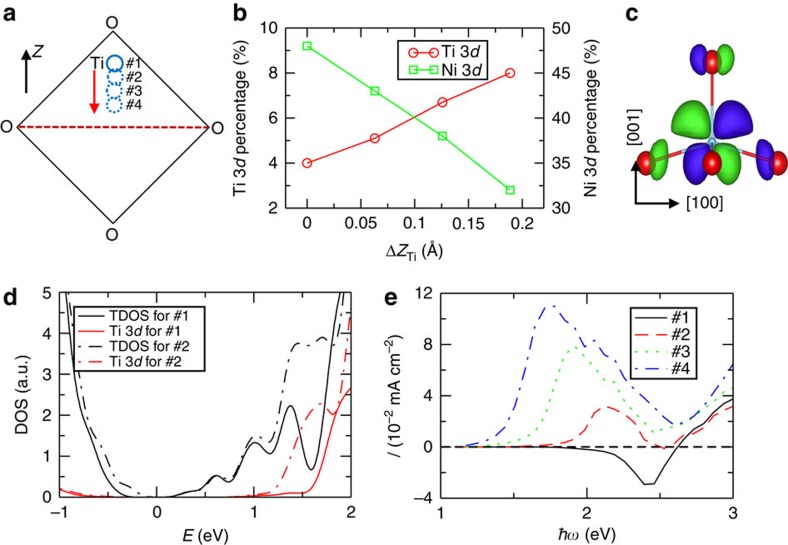
The photocurrent enhancement by artificial local structure displacement and the corresponding change of the electronic composition. (**a**) The schematic representation of how the Ti atoms are moved with respect to the fully relaxed structure (structure #1), (**b**) the percentages of the Ti and Ni 3*d* orbitals at the CB 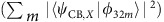 at *X k* point as a function of the antiparallel displacement (Δ*Z*_Ti_) of Ti atoms, (**c**) the orbital character of the Ti–O orbital overlap at the CB, (**d**) total and projected density of states and (**e**) photocurrent (assuming for the 1,000 W m^−2^ light absorption), of the 1 × 1 × 3-layered PNT. The Ti atoms are moved antiparallel to the polarization, reducing but not entirely eliminating their off-centre displacements. The inset label #*n* in **a**,**d** and **e** denotes the structures with different Ti antiparallel displacements. For example, #1 denotes the fully relaxed structure and #2, #3 and #4 denote the structures with Ti atoms displaced antiparallel by 0.06, 0.12 and 0.18 Å, respectively, with respect to their positions in #1. Note that the Ti off-centre displacements in #1 are much larger than those in plain PbTiO_3_. In **b** and **c**, when Ti moves towards the O_4_ plane, the Ti–O interaction becomes more of nonbonding and less of antibonding character, moving the Ti 3*d* orbitals downward and enhancing their contribution to the band-edge electronic states. Also see Fig. 3a for the Glass coefficient change under artificial Ti atom antiparallel displacement, and Supplementary Fig. 2 for the photocurrent change under artificial Pb atom displacement in the rock salt cation arrangement.

The relationship between the CBM wavefunction character, delocalization and shift current sign suggests that eliminating or weakening the differences between the *X* and *R* point wavefunctions can largely eliminate the cancellation of counter-propagating currents and substantially increase the total BVPE response. To verify this, we engineer the CBM wavefunction by changing the chemical bonding in PNT. The bottom of the CB is composed of the Ni 3*d*, Ti 3*d*, Pb 6*p* and O 2*p* orbitals, with the Ni 3*d* orbitals making a major contribution (Supplementary Fig. 1). Since the peak of the Ti 3*d* orbitals is located at a higher energy than that of the Ni 3*d* peak, the contribution of the Ti 3*d* states to the band-edge electronic transitions can be enhanced by a downshift of the Ti 3*d* orbital energy at the CB (Fig. 2d). A reduced overlap between the Ti 3*d* and O 2*p* orbitals would give rise to a weaker Ti–O bond and a smaller energy level difference between the bonding (valence band) and antibonding (CB) Ti–O orbitals, resulting in a downshift of the CBM Ti 3*d* orbitals relative to the those of the relaxed structure^30^,^31^ Therefore, to shift the Ti 3*d* states to lower energies, we increase the Ti–O distances in 1 × 1 × 3 PNT by moving Ti atoms antiparallel to the overall polarization, reducing, but not entirely eliminating the local Ti off-centre displacements found in the relaxed structure (Fig. 2).

**Figure 3:**
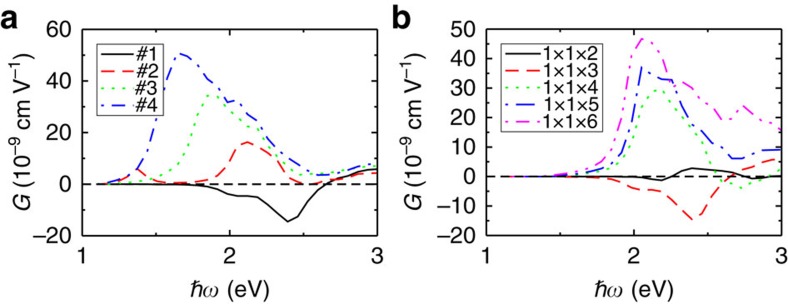
The substantial enhancement of the Glass coefficient. The Glass coefficients of (**a**) the 1 × 1 × 3 superlattice with different Ti antiparallel displacements and (**b**) the 1 × 1 × *N* superlattices. The inset label #*n* in **a** denotes the structures with different Ti antiparallel displacements. For example, #1 denotes the fully relaxed structure (no artificial antiparallel displacement) and #2, #3 and #4 denote the structures with Ti atoms displaced antiparallel by 0.06, 0.12 and 0.18 Å, respectively, with respect to their positions in #1. Note that the Ti off-centre displacements in #1 are much larger than those in plain PbTiO_3_.

The resulting changes in the Ti–O bonding have three effects. First, analysis of the CBM density of states shows that the contribution of the Ti 3*d* (and also Pb 6*p*, not shown) orbitals increases while the proportion of the Ni 3*d* orbitals decreases steadily with Ti atomic movements (Fig. 2b), confirming that CBM electronic structure can be effectively controlled by changes in the local structure and Ti–O bonding. Second, the calculated bandgap decreases, leading to smaller photocurrent onset energy with greater artificial Ti sublattice displacement antiparallel to the polarization. Third, as the Ti sublattice is moved, this first leads to a change in the sign of the shift current direction, followed by a substantial enhancement of the shift current magnitude (Figs 2e and 3a). Following Planck's law of black-body radiation for solar power distribution over light frequency at 6,000 K, the total photocurrent is enhanced by a factor of 97, compared with the ground-state structure. This demonstrates that fairly gentle changes in the structure can significantly affect the CBM character and lead to a markedly enhanced BPVE response.

### Photocurrent enhancement via nanolayering

The design principle of greater CBM delocalization elucidated by the study of artificial lattice adjustment can be realized in nanoscale heterostructures. They are essentially PbNiO_2_-doped PbTiO_3_, but can be viewed as a nanolayer heterostructure of PbNiO_2_ and PbTiO_3_. While a more rigorous temperature-dependent thermodynamic analysis is required for investigating its experimental viability, our 0 K density functional theory (DFT) stability analysis shows that these structures are very likely experimentally accessible in the Pb(Zr_0.2_Ti_0.8_)_0.7_Ni_0.3_O_3−*δ*_ and KBNNO materials for which PNT serves as a prototype. We find that nanolayered structures are preferred to the rock salt-ordered PNT and that under 1% in-plane compressive strains, the 1 × 1 × 2 superlattice with apical O vacancies is more stable than its equatorial counterpart. Thus, the enhancement of the photovoltaic response found by our calculations can be realized through the growth of an epitaxially strained thin film or by layer deposition methods.

**Figure 4:**
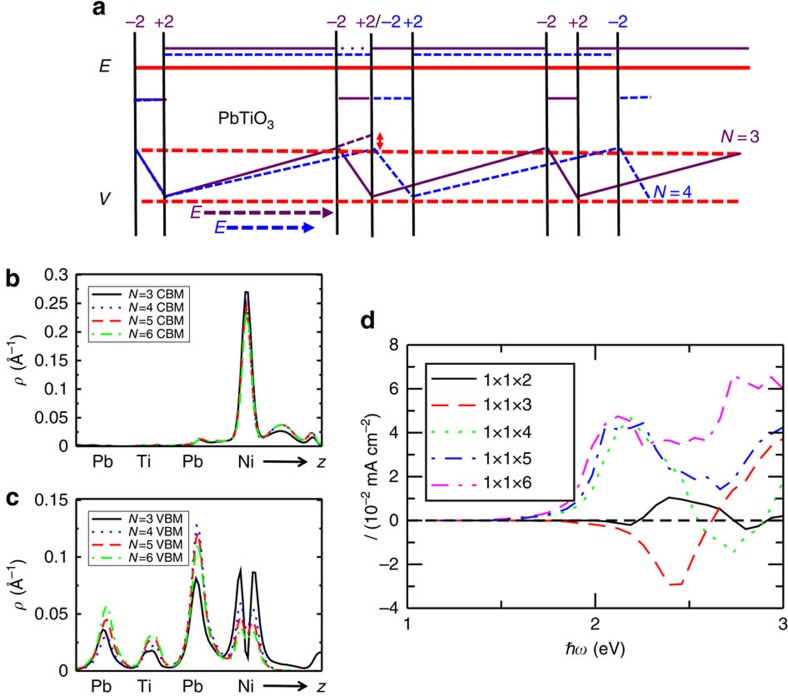
The photocurrent enhancement via nanolayering and the corresponding change of the electric field landscape and state delocalization. (**a**) The electric field and potential inside PNT for *N*=3 and 4. The 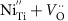 substitution results in adjacent planes of charge. As *N* increases, less screening over a greater distance is generated in response to the potential changes introduced by this charge separation. The electric field magnitude over the PbTiO_3_ layers is approximately 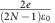, decreasing as *N* increases. (**b**,**c**) The real-space probability density distribution summed over the Cartesian *x* and *y* coordinates 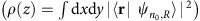 for the CBM and valence band maximum (VBM) states at *R k* point. *N* is the number of layers. For *N*>3, the valence band wavefunction is localized within the supercell, yielding qualitatively distinct behaviour (as seen in Fig. 1c). The decreased electric field allows the wavefunction to decay more slowly, shifting the probability density of the valence band away from nickel, increasing the intracell delocalization. (**d**) The photocurrent (assuming 0.1 W cm^−2^ light absorption) of the layered PNT with varying superlattice periodicity. As *N* increases, the near-gap response stabilizes and the response at 3 eV increases; this represents an across-the-board increase in response per light-absorbing Ni layer due to greater delocalization. Increasing the superlattice *N* reduces the polarizing field on PbTiO_3_, aligning the shift current response across the visible range and increasing the current per absorbed photon.

In PNT, the 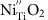 layer has −2 charge, and is adjacent to the 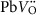 layer with a +2 charge. Thus, 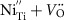 substitution gives rise to charge separation. The Ni-vacancy layers also provide a polarizing field on the PbTiO_3_ layers, with this effect reduced as the Ni-vacancy layers are spaced further apart. Approximating the layers as simple charged sheets, we obtain a periodic series of regions with electric field, analogous to the polar catastrophe in LaAlO_3_/SrTiO_3_ (ref. 18). As the separation between the Ni increases with increasing layer numbers *N*, the electric field in the bulk PbTiO_3_ layers becomes smaller under short-circuit boundary conditions, and bound charges screen the field over a greater length, delocalizing the electron densities (Fig. 4a). This mechanism for the control of wavefunction delocalization and photocurrent is confirmed by the analysis of the electronic structure and the calculated response for 1 × 1 × *N* (*N*=2–6) PNT supercells with one NiO_2_ plane per supercell. To show the changes due to the delocalization, we plot the layer-averaged probability density as a function of layer normal coordinate for the relevant CBM and valence band maximum (VBM) states (Fig. 4b,c). Going from *N*=3 to 4, these states, especially the valence band state, become more delocalized, corresponding to the enhancement of the shift current magnitude for a certain photon energy. In addition, this delocalization is only moderately enhanced from *N*=4 to 6, resulting in the relatively flat progression of shift current at a particular photon energy observed for *N*>3.

Comparison of the calculated shift current spectra shows that contrary to naive expectations, reduction of the Ni fraction actually increases the effectiveness of substitution, increasing the response and reducing the bandgap as the number of layers is increased (Figs 3b and 4d). This enhancement of the total photocurrent is due both to the enhancement of photocurrent magnitude at a certain photon energy and to the alignment of the photocurrent sign at different photon energies throughout the spectrum. The effect for visible-light illumination is quite marked, with an increase by a factor of 43 due to a simple insertion of additional bulk PbTiO_3_ layer(s). Such extraordinary sensitivity underscores the importance and usefulness of nanoscale ordering for the BPVE engineering. We find that the total shift current under visible-light illumination is also over 10 times greater than that for the BiFeO_3_ material that has been the standard for experiments on the photoresponse of ferroelectrics. The proximate reason for the enhancement in response becomes clear on looking at the response along the *X*–*R* line (Fig. 1d and Supplementary Fig. 3). The difference between the 1 × 1 × 3 and 1 × 1 × 4 is due to the disappearance of the sign change versus *k* point in the shift vector and the resulting shift current response. The shift current magnitude then further increases with the number of PbTiO_3_ layers. As *N* increases further, the effect of eliminating cancellation of counter-propagating photocurrents at different photon energies starts to dominate, and this further enhances the total photocurrent for the whole visible-light spectrum.

## Discussion

The nanoscale layered heterostructures have another potential benefit. Recent experimental work has highlighted the importance of conventional transport characteristics for BPVE^7^ Essentially, the performance of the bulk photovoltaic materials depends not only on their current generation capability but also on the photovoltage that they can sustain. This latter quantity is determined by the resistance of the current leaking back through the material, and the design of useful devices will therefore require control over conventional conductivity. In BiFeO_3_, domain walls can serve as barricades against such leakage, substantially increasing the resistance and, consequently, the maximum photovoltage. We note that the present system similarly features a nanoscale heterogeneous layered structure and may be viewed as a nanoscale composite with alternating photocurrent-generating layers and insulating layers, ideal for a BPVE device.

The foregoing holds important lessons for engineering bulk photovoltaic materials. First, it indicates that the problems of bandgap reduction and state delocalization can be partially separated. Nickel substitution provides lower-energy CB states that tend to be localized along the layer normal direction and are not favourable for a large shift current. Nevertheless, these states may participate in transitions with what are essentially PbTiO_3_ states that are delocalized and thus favour larger shift currents. We have chosen a material with robust shift current potential but a large gap (PbTiO_3_), and introduced a modification that solves the latter without losing the former. Second, it emphasizes the importance of not only the magnitude of photocurrent as a factor in response, but consistency in direction, which is sensitive to relatively small structural changes, and therefore amenable to manipulation. Third, the electrostatic effects of the 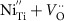 substitution on the electronic structure of bulk PbTiO_3_ are pronounced and most apparent for lower nickel fractions. We hope that our work will motivate further studies of photoresponse in nanoscale ferroelectrics.

The fact that a substantial enhancement of the BPVE is found in an ordered nanostructure highlights the importance of order/disorder effects in photoferroelectrics. It has been long accepted that most ferroelectrics and related materials exhibit local deviations from their average structures, whether in the form of disordered *B*-cation displacements in the conventional BaTiO_3_ and KNbO_3_ ferroelectrics, in relaxor ferroelectrics or in solid solutions^20^,^32^,^33^ The disorder in BaTiO_3_ and KNbO_3_ is due to the presence of ordered chains of displacements and that in the relaxor-like material Na_1/2_Bi_1/2_TiO_3_ is related to planar defects, and both of these structural features are found in our system^19^,^32^ This order/disorder scenario leads to intriguing physics of electronic structure, phase transition, ferroelectricity, piezoelectricity, ferroelasticity, magnetism, and, in our case, the ferroelectric photovoltaic effect. Therefore, to obtain a good ferroelectric photovoltaic, attention should be paid to the effects of order/disorder and to the manipulation of the electric field landscape and bandgap via ordering/disordering; the present work serves as a good starting point for this approach.

Finally, we point out that our finding is not specific to the particular system studied here, but is rather general to a variety of ferroelectric oxide systems incorporating Ni (for example, PbNiO_2_-doped PZT and KBNNO) and other 3*d* transition metals. This is because the contributing electronic states in such a complex system are typically mixtures of different atomic orbitals that can be tailored by changes in structure and electrostatics, and by ordering/disordering. However, due both to the complexity of the relationship between photocurrent, electronic structure and atomic structure, and to the current paucity of visible-light-absorbing ferroelectric photovoltaic materials, a careful and detailed analysis will be required to estimate the magnitude of the nanolayering effects for each particular system.

## Methods

### First-principles calculation

A previously developed approach implemented in our in-house code that yields good agreement with experiment for shift current magnitude and spectral profile was used to calculate the shift current^17^,^34^ The QUANTUM-ESPRESSO code was used to perform DFT calculations with the local density approximation functional^35^,^36^,^37^ All elements are represented by norm-conserving, optimized nonlocal pseudopotentials^38^ For structural optimizations, Monkhorst–Pack grids^39^ with at least 6 × 6 × 6 *k*-point meshes were used, while for the self-consistent and non-self-consistent calculations, finer *k*-point grids up to 40 × 40 × 40 were used to get a well-converged shift current response. The DFT+*U* method was used to improve the description of *d*-orbital electrons, with the Hubbard *U* parameterized by the linear-response approach^40^ The calculated *U* values are ≈4.7 and ≈8.9 eV for Ti and Ni, respectively. All photon energies are shifted by 1.05 eV so that PbTiO_3_ is at its experimental bandgap (3.6 eV). Heyd-Scuseria-Ernzerhof hybrid functional calculations^41^ were also used to corroborate the bandgap trends obtained by DFT+*U* calculations.

### The shift current susceptibility tensor

Tetragonal PbTiO_3_ belongs to the *P*4*mm* space group, corresponding to the *C*_4*v*_ point group; in this case, the third rank shift current response tensor must have the form


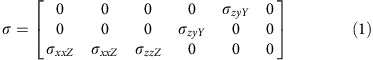


where the lower- and upper-case letters represent directions of the light polarization and photocurrent, respectively. The Glass coefficient


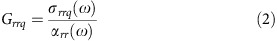


describes the current response in a thick sample and includes the light attenuation effect due to the absorption coefficient *α*_*rr*_(*ω*). We choose our polarization direction to be normal to the Ni layers, so that all analysed systems have the same symmetry properties, and (for unpolarized light) only the *xxZ* and *zzZ* components are relevant. In PNT, the shift current induced by the perpendicularly polarized light (*xxZ*) is much stronger than that induced by the parallel polarized light (*zzZ*) because of their different absorption efficiencies. In the present work, we focus only on the more substantial *xxZ* component.

## Additional information

**How to cite this article:** Wang, F. *et al.* Substantial bulk photovoltaic effect enhancement via nanolayering. *Nat. Commun.* 7:10419 doi: 10.1038/ncomms10419 (2016).

## Supplementary Material

Supplementary Information Supplementary Figures 1-3

## Figures and Tables

**Table 1:** Comparison of the photocurrent and electronic properties of different compositions of PNT.

**Structures**	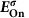	***σ***_**Max**_	***G***_**Max**_	***P***	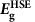	**Δ*****E***
	**(eV)**	**(10**^**−4**^** V**^**−1**^**)**	**(10**^**−9**^** cm V**^**−1**^**)**	**(C m**^**−2**^**)**	**(eV)**	**meV**
PbTiO_3_	3.6	3.7	12.0	0.81	2.71	—
1 × 1 × 2	2.1	1.5	3.6	0.73	1.58	0
Rock salt	2.1	6.1	11.0	0.96	2.05	+30
1 × 1 × 3	1.9	3.9	14.0	0.91	1.58	—
1 × 1 × 4	1.6	4.7	29.7	1.10	1.33	—
1 × 1 × 5	1.5	4.5	36.9	1.07	1.28	—
1 × 1 × 6	1.5	6.7	46.8	1.05	1.08	—

Calculated shift current onset energy 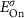, maximum shift current susceptibility *σ*_Max_, maximum Glass coefficient *G*_Max_, polarization *P* and Heyd-Scuseria-Ernzerhof (HSE) hybrid functional bandgap 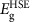 of the layered and rock salt PNT. Δ*E* (meV per atom) is the relative stability of different cation arrangements that have the same composition. PbTiO_3_ values are also shown for comparison. Maximum values are for energies smaller than 3.0 eV (*E*<4.0 eV for PbTiO_3_).
