# Supplementary material for: Substantial bulk photovoltaic effect enhancement via nanolayering
Source: Nat Commun. 2016 Jan 21;7:10419. doi: 10.1038/ncomms10419 (PMC4735945; doi:10.1038/ncomms10419)
Supplement: Supplementary Information — Supplementary Figures 1-3 [file ncomms10419-s1.pdf]

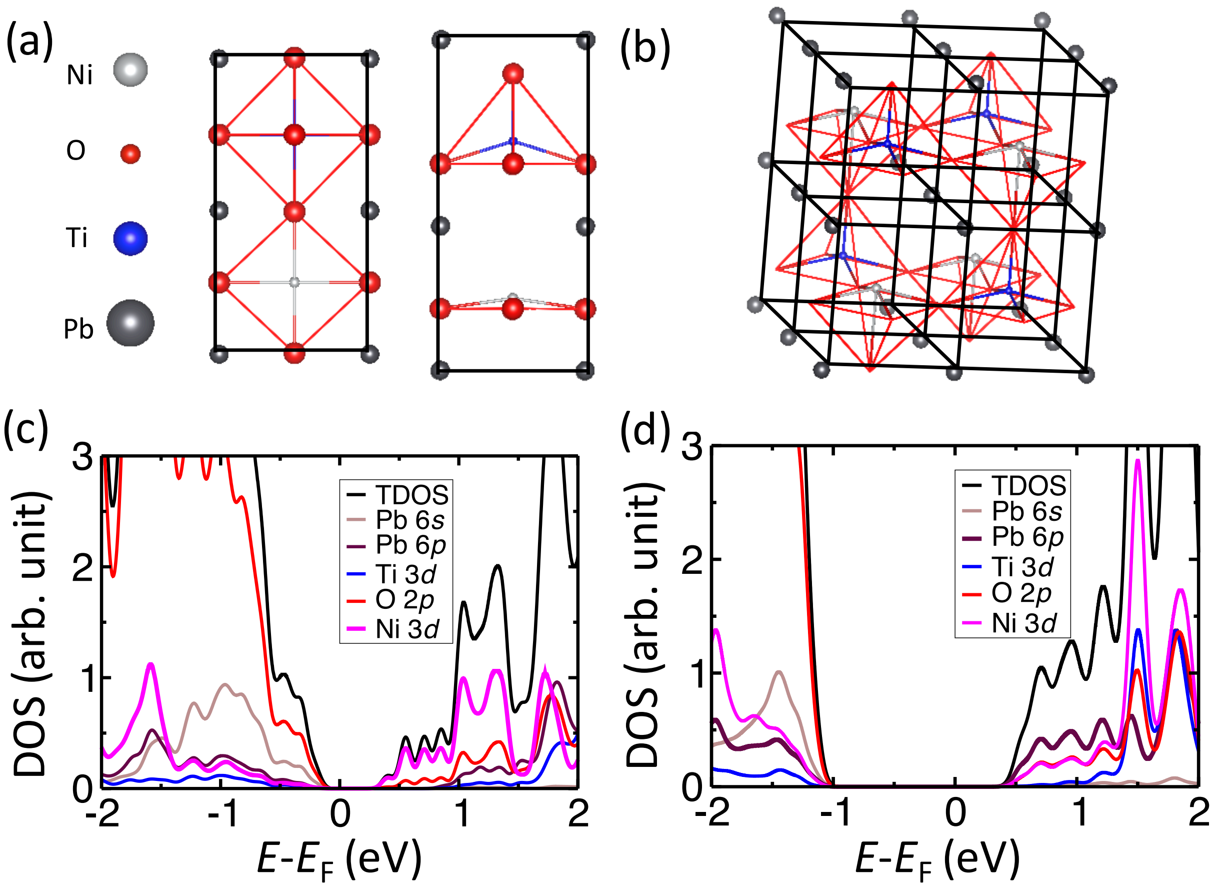

Supplementary Fig. 1. The schematic atomic structure and density of states (DOS) of PNT. The atomic structures of (a) $1 \times 1 \times 2$ layered and (b) rocksalt PNT with apical O vacancies. The atomic structure of the $1 \times 1 \times 2$ layered structure with equatorial O vacancies is also shown in (a). The DFT+U DOSs of (a) $1 \times 1 \times 2$ layered and (b) rocksalt PNT with apical O vacancies. Note that Ni 3d orbitals make a major contribution to the lower conduction band (CB) in the layered structure while Pb 6p orbitals contribute the most to the CB of the rocksalt structure.

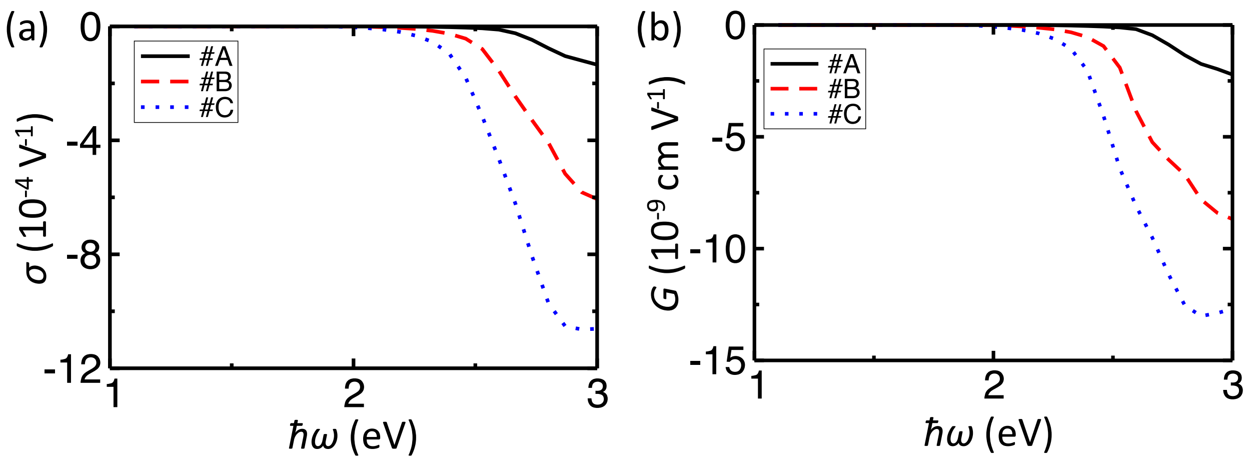

Supplementary Fig. 2. The enhancement of the shift current magnitude in the rocksalt PNT by artificial Pb atom movement. The (a) shift current susceptibilities (σ_{xxZ}) and (b) Glass coefficients (G_{xxZ}) of rocksalt PNT with different Pb displacements. #B is the fully relaxed structure, while #A and #C are the structures with Pb atoms moved parallel and antiparallel to the polarization direction by 0.12 Å with respect to their positions in #B. The photocurrent response can be substantially enhanced when Pb atoms are moved antiparallel to the polarization direction. This is due to the decrease of the Pb (6p)-O antibonding interaction, leading to a lower energy position of the Pb 6p orbitals at the conduction band. This reduces the contribution to the band-edge shift current from Ni 3d orbitals while increasing that from the more extended Pb 6p orbitals.

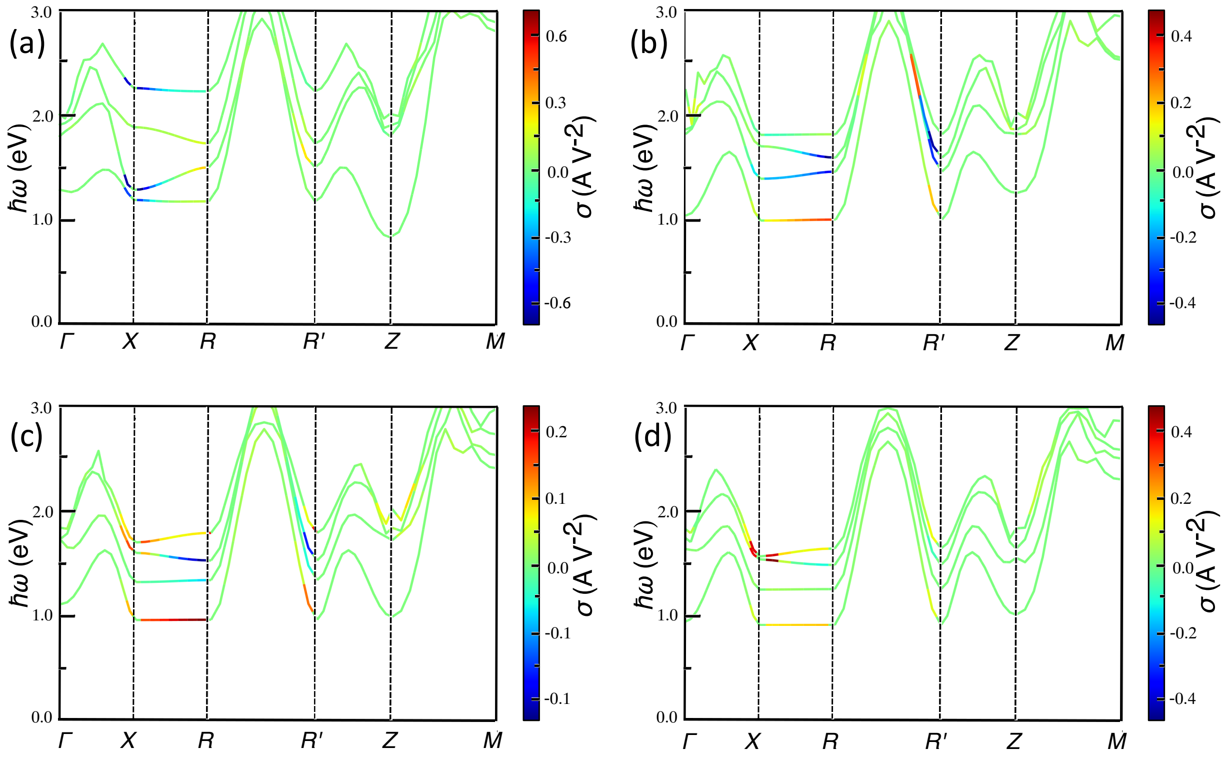

Supplementary Fig. 3. Mapping of the shift current strength at different transition energies for the Brillouin zone of the $1 \times 1 \times N$ layered PNT [N=3, 4, 5, and 6 for (a), (b), (c), and (d), respectively]. R' indicates R plus a reciprocal lattice vector, so that $R \to R'$ traverses the Brillouin zone. The color gives the value of the photocurrent response σ (A V^{-2}). For all four compositions, the band-edge shift current mainly arises from electronic transitions at near X and R regions, particularly at the k points along the X-R line. For N=3, the shift current direction at near X point is opposite to that at near R point, so that their contributions to the total shift current counteract each other. However, for N >4 the shift currents at the near X and near R points are in the same direction, and they add up with each other constructively, giving rise to an enhancement of the total shift current.
